# Cardioprotection of mAb2G4/ODN/lip on Myocardial Ischemia-Reperfusion Injury via Inhibiting the NF-*κ*B Signaling Pathway

**DOI:** 10.1155/2023/5034683

**Published:** 2023-04-27

**Authors:** Zujin Xu, Zhuoran Wei, Yali Zhu, Guoqing Jing, Liufang Chen, Jia Zhan, Yun Wu

**Affiliations:** Department of Anesthesiology, Zhongnan Hospital of Wuhan University, 169, East Lake Road, Wuhan 430071, China

## Abstract

Substantial evidence suggests that the interventions of NF-*κ*B would likely effectively prevent inflammatory response and reduce myocardial damage in the ischemic myocardium. And the NF-кB decoy ODN is a specific inhibitor that suppresses the expression of NF-*κ*B. Herein, we revealed the effect and possible mechanism of mAb2G4/ODN/lip on myocardial ischemia-reperfusion injury (MI/RI). As shown in the results, post-treatment with mAb2G4/ODN/lip improved the impaired histological morphology in the MI/RI model and elevated cell viability in the H/R model. The mAb2G4/ODN/lip complex inhibited the NLRP3 signaling pathway and decreased the expression of LDH, IL-1*β*, TNF-*α*, IL-6, and MDA. Mechanistically, we demonstrated that post-treatment with mAb2G4/ODN/lip exerted protective effects against I/R injuries by inhibiting the NF-кB-related inflammatory response. In summary, the present study may offer a novel therapeutic strategy for treating MI/RI.

## 1. Introduction

Myocardial ischemia-reperfusion injury (MI/RI) is currently a subject of immense disease and medical interest all over the world. MI/RI refers to the definition that severer injuries would occur to the original ischemic myocardium after the restoration of the blood supply [1, 2]. Multiple pathological processes such as severe inflammation [3], excessive reactive oxygen species accumulation [4], intracellular calcium overload [5], and apoptosis [6] contribute to the development of MI/RI. In recent years, much research shed light on the effects of NF-*κ*B-mediated inflammation in MI/RI [7–9]. Once activated, NF-кB leads to the severe secretion of various inflammatory factors, which further attract multiple inflammatory cells such as neutrophils and macrophages. And the prominent infiltration of inflammatory cells leads to severe inflammatory damage to myocardial tissue [10, 11]. Gul-Kahraman et al.'s team found that reduced melatonin synthesis aggravated the age-related cardiovascular disease. Mechanistically, they uncovered that melatonin could reduce MI/RI-induced myocardial injury by regulating the NF-*κ*B signaling pathway [12]. Therefore, activation of NF-*κ*B may be critical in the onset and development of MI/RI. As a single-stranded DAN short fragment, NF-кB decoy oligodeoxynucleotide (ODN) has high similarity to the NF-кB-binding DAN fragment, thereby inhibiting the transcription of targeted genes [13]. Remarkably, the NF-кB decoy ODN was successfully applied to treat various inflammatory diseases in the animal model, such as rat periapical lesion model [14], murine inflammatory bowel disease model [15], and myocardial ischemia-reperfusion injury model [16]. However, the application of ODNs has limitations due to some obstacles such as a short half-life, rapid digestion by endocytosis and nucleases, and poor cellular uptake [17]. Cationic liposomes, defined as artificial microscopic vesicles, protect target molecules from digestion by incorporating anionic materials through static electronic interaction [18]. Given their ability to encapsulate nanodrugs and nucleic acids, liposomes had been widely applied in the delivery of decoy ODNs. And related studies demonstrated that NF-*κ*B decoy ODN complex with cationic liposomes exerts more significant anti-inflammatory effects [19, 20]. Despite the increased anti-inflammatory effects of ODN/lip complex, there is still a lack of targetability to the injured myocardium. KO et al.'s team once used a mAb2G4-modified vector to translocate the target gene into ischemic-injured cardiomyocytes. Intriguingly, the results demonstrated that the transfection efficiency of target genes significantly enhanced in ischemic-zone cardiomyocytes [21]. Hence, we hypothesized whether ODN and mAb2G4 could be combined into a complex to target damaged myocardia and achieve more significant therapeutic effects. In the present study, we constructed a myocardial ischemia-reperfusion model in rats by reperfusion treatment after ligation of the left anterior descending branch of the coronary artery. Strikingly, the present results effectively support the hypothesis we mentioned above.

## 2. Materials and Methods

### 2.1. Animals

We declare that all experiments in the present article were approved by the Medical Ethics Committee of Zhongnan Hospital of Wuhan University. Male Sprague-Dawley rats (3 to 4 months, 250 ± 10 g, Experiment Animal Center of Wuhan University) were used for the current study. All animals were housed in a controlled environment with a 12-hour light/dark cycle at 23 ± 3°C and 40–70% humidity and allowed free access to food and water before the experiment. All animals received human care following the Guide for the Care and Use of Laboratory Animals (8th ed., Washington DC: National Academies Press (US), 2011).

### 2.2. Drugs and Reagents

0.25% trypsin (15050065), fetal bovine serum (10099-141), and DMEM (12800-058) were purchased from Gibco, USA. DSPE-PEG (2000) amine, DSPE-PEG (2000) PDP, and DSPC were purchased from Avanti, USA, DTT. Evans blue dye was bought from BioFroxx, Germany. 2,3,5-Triphenyl tetrazolium chloride (TTC) was purchased from Aladdin Biochemical Technology Co., Shanghai. SPDP was purchased from Sigma, USA. Rabbit anti-NF-*κ*B polyclonal antibody was purchased from BioWorld, USA. Lipofectamine™ 2000 (11668-019) was purchased from Invitrogen, USA. MTT cell proliferation and toxicity assay kit (KGA312) was obtained from Nanjing KGI Biological Co. MDA assay kit and ELISA kits of LDH, IL-1*β*, TNF-*α*, and IL-6 were products from Nanjing Jiancheng Institute of Biological Engineering. Mouse anti-myosin monoclonal antibody was the product from Abcam, UK, and rabbit anti-NF-*κ*B, anti-NF-*κ*B p65, anti-*β*-actin, and anti-mouse IgG polyclonal antibodies (1 : 1000) were purchased from BioWorld, USA.

### 2.3. Preparation of ODN-lip and mAb2G4/ODN/lip Complex

Sequences of NF-*κ*B decoy ODN containing both the specific p50 (GGGAC) and p65 (TTCC) *κ*B binding sites were as follows: 5′-AGT TGA GGG GAC TTT CCC AGG C-3′ and 3′-TCA ACT CCC CTG AAA GGG TCC G-5′. ODNs were synthesized by GenScript Co., Ltd. (Nanjing, China). Preparation of ODN-lip: ODN-lip was prepared using the film hydration method. 5 ml of chloroform was dried to a thin film under reduced pressure after dissolving 5 *μ*mol DSPC, 4 *μ*mol cholesterol, 0.25 *μ*mol DSPE-PEG (2000) amine, and 0.05 *μ*mol DSPE-PEG (2000) PDP (molar ratio of 50 : 40 : 2.5 : 0.5). 1 ml PBS solution was added at a concentration of 200 *μ*g/ml NF-*κ*B ODN to dissolve lipid membranes, blending for 45 min at room temperature to hydrate and swelling of the film. The resulting suspension was then subjected to vortex turbulence and sonication, followed by 450 nm, 220 nm, and 80 nm microporous membranes. Disposing the recovered liposomes with DNase and Exonuclease III for 1 hour at 37°C, EDTA was used to terminate the reaction. The mixture passed through a Sephadex G-100 column to separate liposomes encapsulating DNA fragments from DNA degraded by nuclease. Sulfhydrylation of antibody: the rat cardiac myosin, mAb2G4 (Abcam, Cambridge, UK), at the concentration of 1 mg/ml was mixed with SPDP solution at the concentration of 1.25 mg/ml and 25°C for 30 min to generate PDP-IgG. The reacted mixture passed through a Sephadex G-25 column to remove excess SPDP and other by-products. DTT was added to collect PDP-IgG to make a final concentration of 50 mmol/L, and the mixture reacted with stirring at 25°C for 25 min. They passed through a Sephadex G-25 column to remove excess DTT and obtain the thiolated rat cardiac myosin antibody (IgG-SH). Preparation of mAb2G4/ODN/lip complex: the collected IgG-SH immediately was mixed with liposomes (molar ratio of 1 : 1000), stirred overnight at room temperature in the dark, and then passed through Sepharose CL-4B column to wipe off unbound IgG-SH, eventually achieving the immune liposomes combined with cardiac myosin monoclonal antibody.

### 2.4. In Vivo Induced Myocardial Ischemia-Reperfusion Injury in Rats

The myocardial I/R model was established as previously described [22, 23]. After being anesthetized with an intraperitoneal injection of pentobarbital sodium (50 mg/kg), the rats were attached to electrocardiograph probes to produce an electrocardiogram (ECG) image. Tracheostomy tube ventilator-assisted breathing was used and mechanically ventilated with oxygen-enriched room air with a DW 2000 animal breathing machine (VT = 2–3 ml/100 g, RR = 80 − 90 bpm, and I : E = 1 : 2). Throughout the experiment, the rats were placed on a heating mat to maintain normothermia (35°C). The right femoral vein was cannulated for fluid and drug transfusion. The chest was opened via a left thoracotomy between the third and the fourth costal bones. After the pericardium was incised, a thoracic spreader was used to expose the thoracic cavity. A 6-0 atraumatic suture crossed underneath the left anterior descending coronary artery (LAD), and a small plastic tube was placed in the zoom between the suture and LAD. A reversible LAD occlusion model was formed by fastening the ends of the tie. After a stabilization period of 10 min, the LAD was ligated for 30 min followed by reperfusion for 120 min. The occurrence of changes from red to pale in the myocardium supported by the blocked coronary artery and of elevation of the ST segment on ECG indicated that the occlusion of the LAD was achieved. Adequate reperfusion was confirmed by the observation of epicardial hyperemia, and more than 50% reversion of ECG changes is caused by ischemia. After a 120 min reperfusion followed by the infusion of the drugs, all animals were sacrificed, and the blood samples were collected from the right ventricle.

### 2.5. Experimental Protocol In Vivo

**Figure 1:**
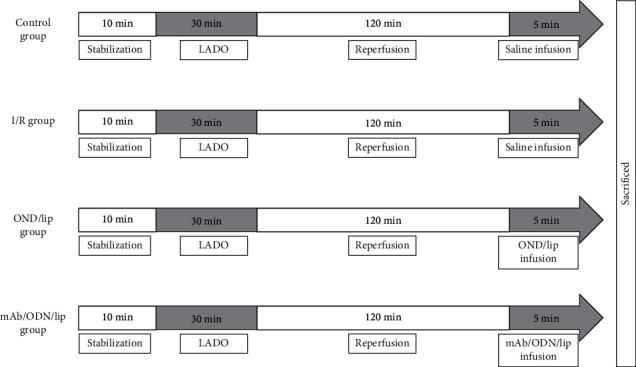
The experimental protocol in vivo. LADO: left anterior descending coronary artery occlusion; I/R myocardial ischemia-reperfusion; ODN/lip: liposome-NF-*κ*B decoy oligodeoxynucleotide complexes; mAb2G4-ODN-lip: monoclonal anti-myosin antibody 2G4-attached cationic liposome-NF-*κ*B decoy oligodeoxynucleotide complexes. All animals were sacrificed in the end of the experiment.

Male adult Sprague-Dawley rats were randomly divided into four groups (*n* = 8 for each): control group (rats underwent shame operation without LAD ligation and drug treatment), I/R group (LAD occlusion for 30 min and then reperfusion for 120 min), ODN-lip group (the ODN at dose of 600 *μ*g/kg, ODN-lip continuous infusion via the right femoral vein at least 5 min once successful occlusion, followed by reperfusion for 120 min), and mAb2G4/ODN/lip group (the ODN at dose of 600 *μ*g/kg, mAb2G4/ODN/lip continuous infusion via the right femoral vein at least 5 min once successful occlusion, followed by reperfusion for 120 min) (Figure 1).

### 2.6. Histological Examination (HE) of Myocardium

Rat myocardium tissue was fixed in a 4% formaldehyde solution to maintain the original morphological structure of the cardiomyocytes. The water in the tissue was gradually removed by using alcohol as a dehydrating agent from low to high concentrations. After xylene treatment, the tissue blocks were immersed in paraffin wax for embedding and cut into flakes (6 *μ*m), which were dried in a 45°C thermostat. After the removal of paraffin by xylene, sections were stained with hematoxylin and eosin. Finally, light microscope (Nikon, Tokyo, Japan) at ×40, ×200, and ×400 magnifications was applied to evaluate the pathological damage to myocardial tissue.

### 2.7. Myocardial Infarct Size Measurements

Myocardial infarction area was measured according to previous studies [24–26]. Six rats from each group were randomly selected, and the coronary arteries were ligated again at the location where they were ligated during ischemia after reperfusion for 2 hours. And 2 ml of 1% Evans blue dye (BioFroxx, Germany) was injected by a catheter placed in the femoral vein. The hearts were rinsed with precooled 0.1 mol/l phosphate buffer (pH 7.4), dried with filter paper, and then placed in the -80°C refrigerator for 30 minutes. Five pieces of myocardium about 1 mm thick were quickly cut crosswise along the long axis of the vertical left ventricle and sequentially immersed in 1% 2,3,5-triphenyl tetrazolium chloride (TTC, Aladdin Biochemical Technology Co., Shanghai) and incubated for 15 min at 37°C in an incubator protected from light. After fixation with 4% paraformaldehyde (Shanghai Chemical Reagent Company) for 12 hours at 4°C, the infarcted myocardium (pale) clearly separated from the noninfarcted myocardium (blue). The infarcted area and noninfarcted area were analyzed using the ImageJ software. Myocardial infarction area is divided by total area as the percentage of infarct size.

### 2.8. Cell Culture

H9c2 cells, derived from rat embryonic ventricular cardiomyocytes, were purchased from Shanghai Bioleaf Biotech Co. Ltd. (Shanghai, China). H9c2 cells were cultured in Dulbecco modified Eagle medium (DMEM, Sigma) containing 10% fetal bovine serum (FBS, HyClone, Logan, UT, USA) and 1% penicillin/streptomycin (*V*/*V*) (Sigma, St. Louis, MO, USA). The medium was stored in a humidified incubator containing 5% CO_2_/95% air at 37°C. The medium was replaced every 2 days, and cells were subcultured for experimental procedures at 80–90% confluence.

### 2.9. In Vitro Establishment of Cardiomyocyte Hypoxia-Reoxygenation Model

The model for hypoxia and reoxygenation was established using the anaerobic method [27]. Briefly, H9c2 cells were placed inside a sealed air-tight container containing an anaerobic (Mitsubishi Gas Company, Tokyo, Japan). The cells were maintained in a hypoxic condition at 37°C for 120 min and then incubated for 60 min at 37°C in a humidified 5% CO_2_ atmosphere. This resulted in a hypoxic atmosphere by absorbing oxygen and generating carbon dioxide.

### 2.10. Experimental Protocol In Vitro

**Figure 2:**
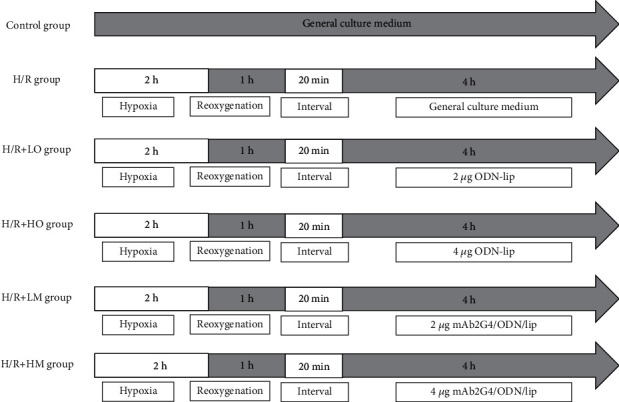
The experimental protocol in vitro. H/R: hypoxia/reoxygenation; LO: ODN/lip at low dose (2 *μ*g); HO: ODN/lip at high dose (4 *μ*g); LM: mAb2G4-ODN-lip at low dose (2 *μ*g); HM: mAb2G4-ODN-lip at high dose (4 *μ*g); interval: transfer to general culture medium.

Cells were divided into six groups according to the random number table method (*n* = 6), control group (cells were cultured in the normal incubating condition), H/R group (cells were subjected to hypoxia for 2 hours and reoxygenation 1-hour treatment), LO group (cells were subjected to hypoxia for 2 hours and reoxygenation 1-hour treatment followed by the treatment with 2 *μ*g ODN/lip), HO group (cells were subjected to hypoxia 2 hours and reoxygenation 1-hour treatment followed by the treatment with 4 *μ*g ODN/lip), LM group (cells were subjected to hypoxia 2 hours and reoxygenation 1-hour treatment followed by the treatment with 2 *μ*g mAb2G4/ODN/lip), and HM group (cells were subjected to hypoxia 2 hours and reoxygenation 1-hour treatment followed by the treatment with 4 *μ*g mAb2G4/ODN/lip) (Figure 2).

### 2.11. Cell Viability Assay

Cell viability was determined by a microculture tetrazolium (MTT) assay kit as described by the manufacturer (M2128-100MG, Sigma), and cells were seeded in 96-well plates at a density of 2 × 104 cells/well. After inducing H/R, the MTT solution (0.5 mg/ml) was added to each well and incubated for 4 hours at 37°C. The medium was removed then, and the metabolized MTT was solubilized with 150 *μ*l DMSO (V900090, Sigma, St. Louis, MO, USA). The absorbance was measured at 490 nm with a microplate reader (MD, SpectraMax 190). The percent viability was defined as the relative absorbance of treated versus that of untreated control cells.

### 2.12. ELISA

The blood samples were collected before the rats were sacrificed. After the H/R procedure, the cell supernatant was collected from the medium. MDA content was determined by MDA assay kit, and the expression of LDH, IL-1*β*, TNF-*α*, and IL-6 in blood samples and cell supernatant was determined via ELISA kits according to the instructions of kits.

### 2.13. Western Blot Analysis

The tissue samples were treated in RIPA lysis buffer at 4°C for 5 min to get 10% homogenates. The homogenates and H9c2 cells were lysed in RIPA buffer at 4°C for 45 min and centrifuged (4°C, 12000 rpm, 10 min), and the supernatant was collected for the next experiment. Protein expression was determined by a bicinchoninic acid (BCA) protein assay kit following the manufacturers' instructions. The sample (40 *μ*g) was subjected to 10% sodium dodecyl sulfate-polyacrylamide gel electrophoresis and transferred to polyvinylidene fluoride membranes. And then, the membrane was blocked in 5% nonfat milk in Tris-buffered saline (TBS) containing 0.1% Tween 20 (TBST) for 120 min at indoor temperature. After washing, primary antibodies of p-p65, p65, and *β*-actin were added and incubated overnight at 4°C, and the membrane was washed and incubated with the corresponding horseradish peroxidase-conjugated secondary antibody at room temperature for 120 min. The protein bands were visualized by an enhanced chemiluminescence system, and the density of each band was quantified by densitometry using Quantity One software (Bio-Rad, Hercules, CA). Detection of *β*-actin was used as a control.

### 2.14. Statistical Analysis

All data were presented as the mean ± standard deviation (SD) of the values obtained from at least 3 independent experiments. The means of different treatments were compared by Student's *t* test or 1-way analysis of variance (ANOVA), followed by Tukey's test as appropriate. *P* < 0.05 was considered statistically significant. All statistical comparisons were performed by GraphPad Prism version 9.1.0 for Windows (GraphPad Software).

## 3. Results

### 3.1. ODN/lip and mAb2G4/ODN/lip Alleviated Pathological Injury of Myocardium Subjected to MI/RI

**Figure 3:**
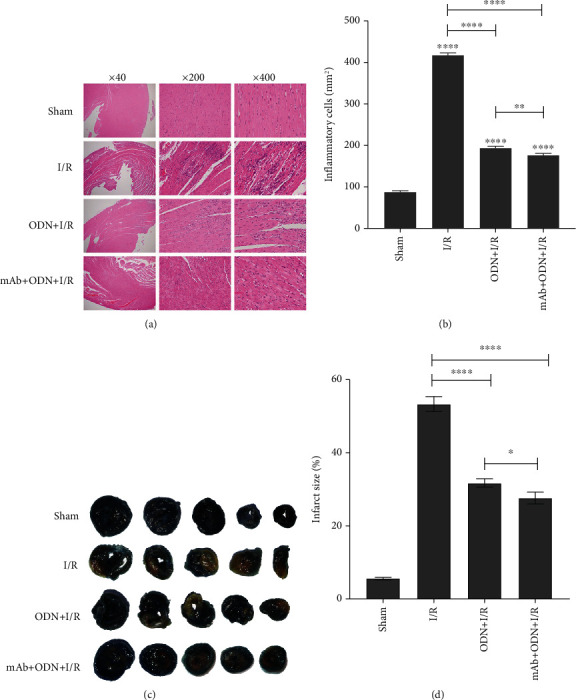
Effect of ODN-lip and mAb2G4-ODN-lip on myocardial histology and infarct size. The results of myocardial histology (a). Inflammatory cells/mm^2^ were counted by ImageJ software (b). The results of the TTC staining (c). The infarct size was calculated by ImageJ software (d). ODN/lip: liposome-NF-*κ*B decoy oligodeoxynucleotide complexes; mAb2G4-ODN-lip: monoclonal anti-myosin antibody 2G4-attached cationic liposome-NF-*κ*B decoy oligodeoxynucleotide complexes. Infarct size = Myocardial infarction area/total area (%). Data were expressed as mean ± S.D. ^ns^*P* > 0.05, ^∗^*P* < 0.05, ^∗∗^*P* < 0.01, ^∗∗∗^*P* < 0.005, and ^∗∗∗∗^*P* < 0.0001. I/R, ODN/lip, and mAb2G4/ODN/lip group vs. control group; ODN/lip group and mAb2G4/ODN/lip group vs. I/R group; ODN/lip group vs. mAb2G4/ODN/lip group.

In the I/R group, cells exhibited noticeable myocardial cell swelling, degeneration, and loss of transverse striations, alongside marked neutrophil infiltration. A significant increase of inflammatory cells was observed, and they microscopically appeared as one predominantly blue-dotted inflammatory cell (mainly neutrophils, lymphocytes, and monocytes) (Figures 3(a) and 3(b)). Compared with the I/R group, myocardial tissue damage was alleviated in the ODN group and mAb2G4 group. And the protective effects on myocardial tissue were more noticeable in the mAb2G4 group. The results of TTC staining also indicated that ODN and mAb2G4/ODN/lip could attenuate tissue damage caused by myocardial ischemia-reperfusion. The percentage of myocardial infarct area in the ODN/lip and mAb2G4/ODN/lip groups was significantly less than that in the I/R group. And mAb2G4/ODN/lip had an advantage over ODN/lip in terms of treatment effect (Figures 3(c) and 3(d)).

### 3.2. ODN/lip and mAb2G4/ODN/lip Could Suppress Inflammatory Response Caused by Hypoxia-Reperfusion Injury

**Figure 4:**
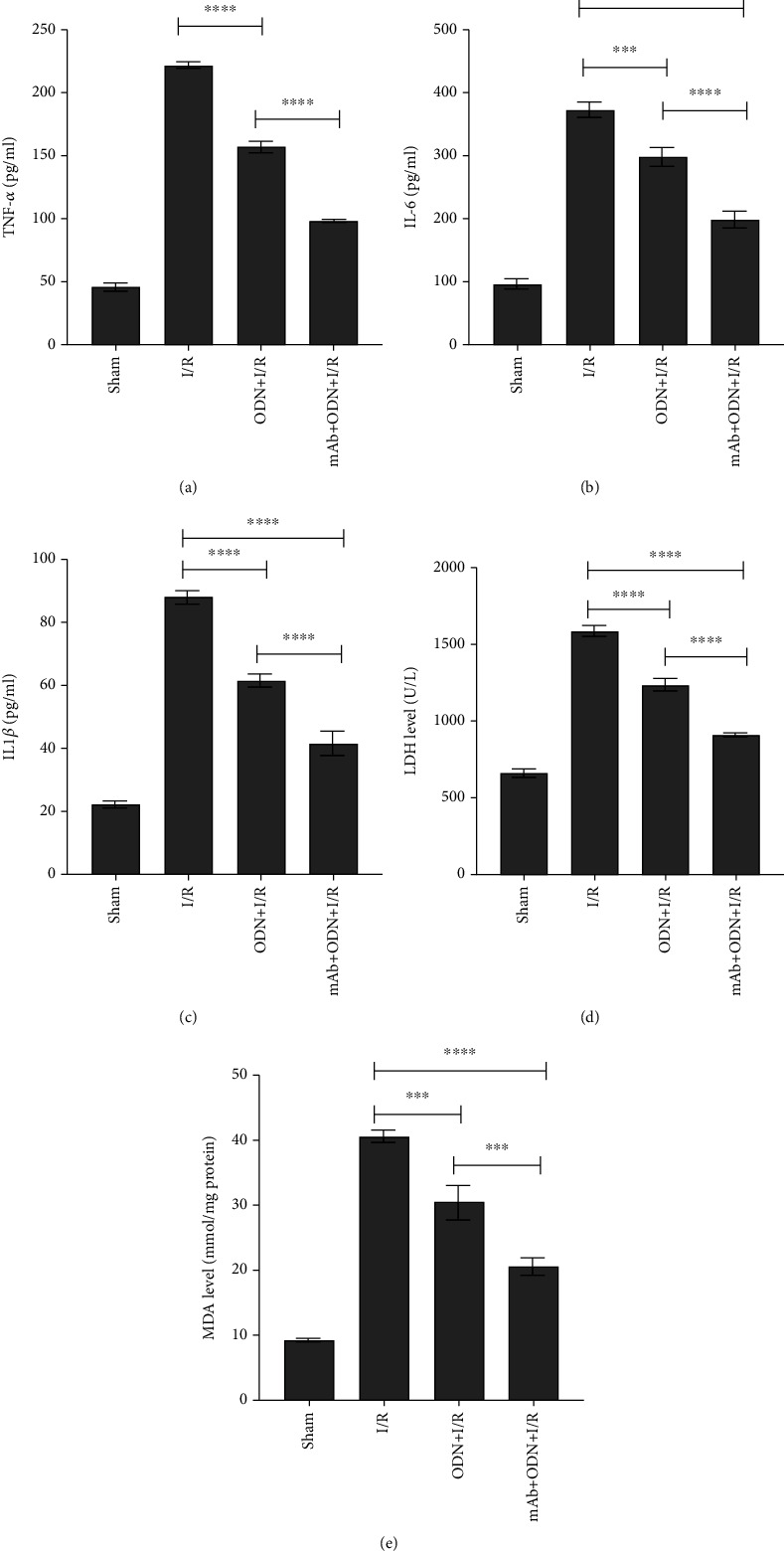
The expression of inflammatory cytokines in vivo experiments. TNF-*α* (a), IL-6 (b), IL-1*β* (c), LDH (d), and MDA (e) in MI/RI rats. Data were expressed as mean ± S.D. ^ns^*P* > 0.05, ^∗^*P* < 0.05, ^∗∗^*P* < 0.01, ^∗∗∗^*P* < 0.005, and ^∗∗∗∗^*P* < 0.0001. ODN/lip group and mAb2G4/ODN/lip group vs. I/R group; ODN/lip group vs. mAb2G4/ODN/lip group.

**Figure 5:**
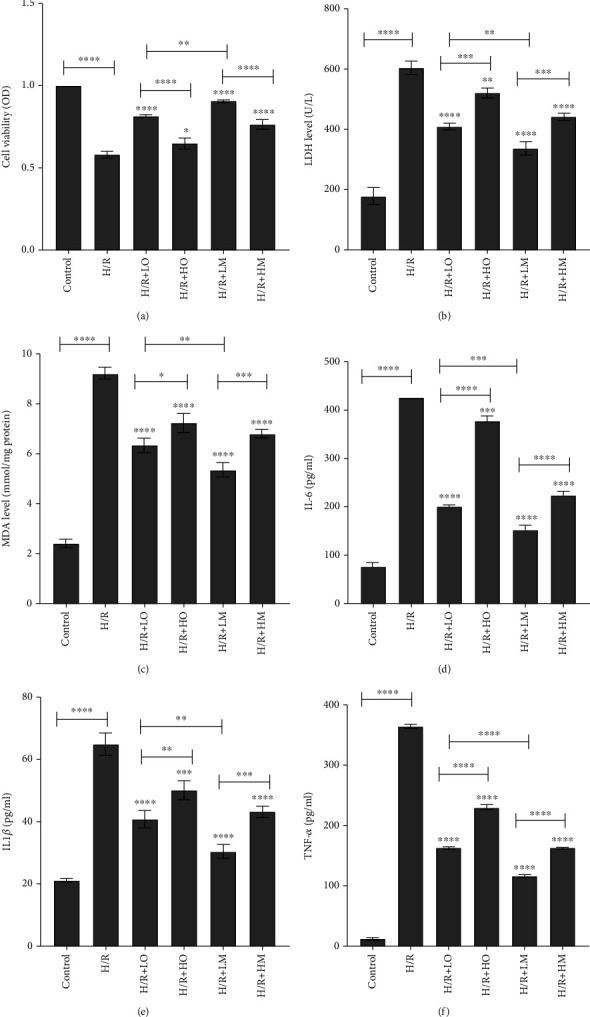
The cell viability and the expression of inflammatory cytokines in vitro experiments. Effects of ODN/lip and mAb2G4/ODN/lip on cell viability (a) and the expression of LDH (b), MDA (c), IL-6 (d), IL-1*β* (e), and TNF-*α* (f) in H/R ventricular myocyte model. Control group (cells were cultured in the normal incubating condition), H/R group (cells were subjected to hypoxia for 2 hours and reoxygenation 1-hour treatment), LO group (treated with 2 *μ*g ODN/lip), HO group (treated with 4 *μ*g ODN/lip), LM group (treated with 2 *μ*g mAb2G4/ODN/lip), and HM group (treated with 4 *μ*g mAb2G4/ODN/lip). Data were expressed as mean ± S.D. ^ns^*P* > 0.05, ^∗^*P* < 0.05, ^∗∗^*P* < 0.01, ^∗∗∗^*P* < 0.005, and ^∗∗∗∗^*P* < 0.0001. H/R group vs. control group; LO group, HO group, LM group, and HM group vs. H/R group; LO group vs. HO group; LM group vs. HM group; LO group vs. LM group.

Blood samples were collected from the right ventricle of each rat following 120 min of reperfusion. ELISA kit and MDA assay kit was used to determine the expression of TNF-*α*, IL-6, IL-1*β*, LDH, and MDA in serum. Results showed that these indicators were significantly upregulated in the I/R group compared with the control group (*P* < 0.0001) (Figures 4(a)–4(e)). Treatment with ODN/lip and mAb2G4/ODN/lip significantly reduced the expression of TNF-*α*, IL-6, IL-1*β*, LDH, and MDA (*P* < 0.0001). And mAb2G4/ODN/lip exerted a more potent effect (*P* < 0.0001) (Figures 4(a)–4(e)). The expressions of LDH, MDA, IL-6, IL-1*β*, and TNF-*α* were significantly upregulated in H9c2 cardiomyocytes after hypoxia/reoxygenation treatment (*P* < 0.0001). Both ODN/lip and mAb2G4/ODN/lip could inhibit the upregulation of these inflammatory factors, and the effect of mAb2G4/ODN/lip was more significant at the same dose (Figures 5(b)–5(f)). In addition, the experimental results also showed that low doses of ODN/lip and mAb2G4/ODN/lip had more pronounced inhibitory effects on inflammatory factors during ischemia-reperfusion myocardial injury. These results indicated that ODN/lip and mAb2G4/ODN/lip could suppressed inflammatory response caused by hypoxia-reperfusion injury no matter in vivo and vitro.

### 3.3. ODN/lip and mAb2G4/ODN/lip Inhibited the Activation of NF-*κ*B/NLRP3 Signaling Pathway in MI/RI Rats In Vivo

**Figure 6:**
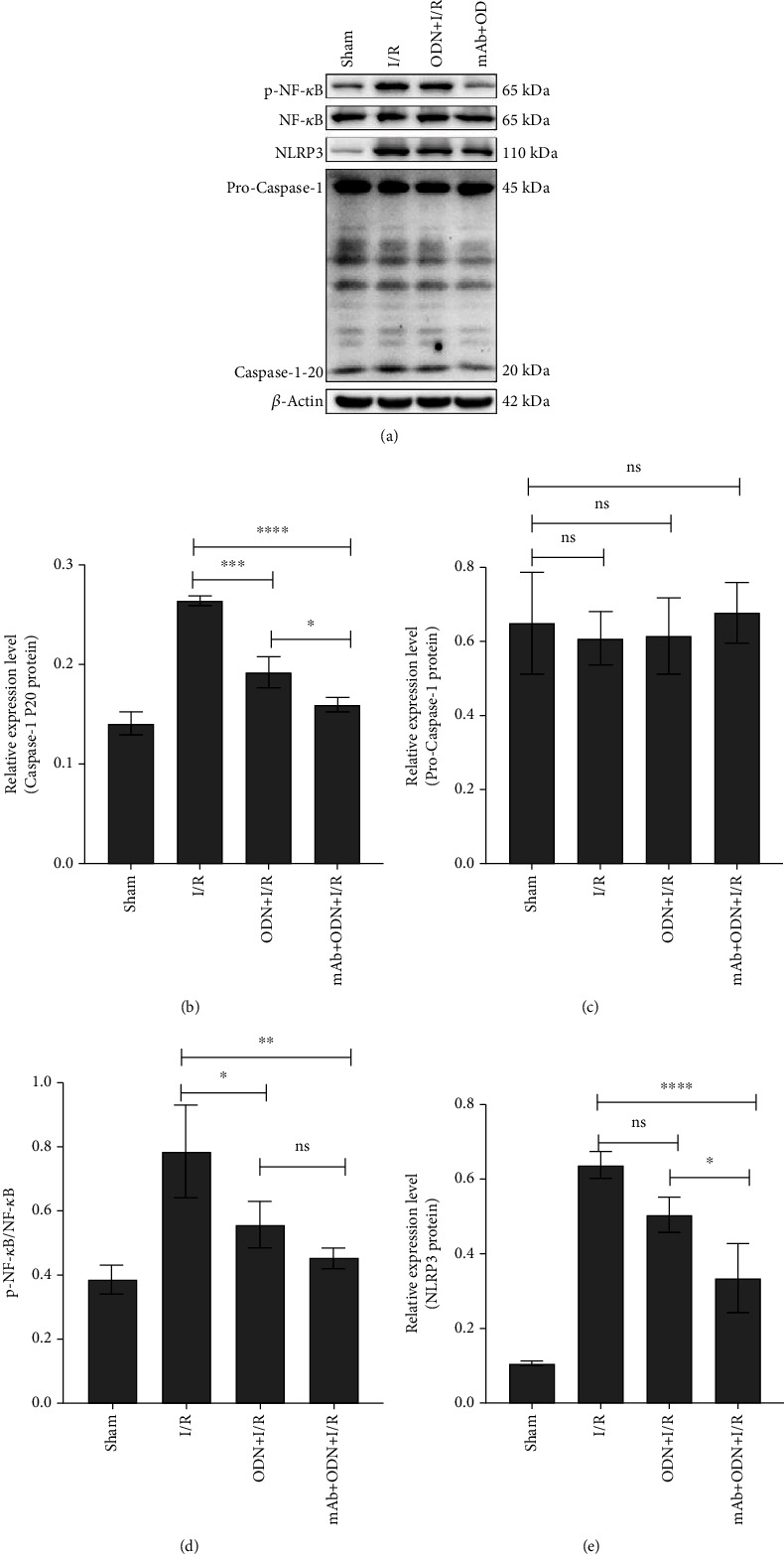
Analysis of the expression of inflammatory proteins by western blot in vivo. Results of western blot in the expression of caspase-1 P20 protein, procaspase-1 protein, p-NF-*κ*B, and NLRP3 protein in vivo (a). Effects of ODN/lip and mAb2G4/ODN/lip on the expression of caspase-1 P20 protein (b), procaspase-1 protein (c), p-NF-*κ*B (d), and NLRP3 protein (e) in MI/RI rat model. Data were expressed as mean ± S.D. ^ns^*P* > 0.05, ^∗^*P* < 0.05, ^∗∗^*P* < 0.01, ^∗∗∗^*P* < 0.005, and ^∗∗∗∗^*P* < 0.0001. ODN/lip group and mAb2G4/ODN/lip group vs. I/R group; ODN/lip group vs. mAb2G4/ODN/lip group.

Rats' heart tissues were obtained for western blot following 120 min of reperfusion. *β*-Actin protein was used as the internal reference protein to detect the expression of some related proteins of NF-*κ*B (Figure 6(a)). According to the results, treatment with ODN/lip and mAb2G4/ODN/lip significantly reduced the expression of caspase-1-P20 protein (*P* < 0.005). And mAb2G4/ODN/lip exerted a more substantial effect than ODN/lip (Figure 6(b)). No significant difference in the expression of procaspase-1 protein among each group was observed (Figure 6(c)). The data suggested that ODN/lip and mAb2G4/ODN/lip inhibited the activation of caspase-1 while did not influence the expression of pro-caspase-1. Treatment with mAb2G4/ODN/lip significantly reduced the expression of p-NF-*κ*B and NLRP3 proteins (*P* < 0.01) (Figures 6(d) and 6(e)). Treatment with ODN/lip reduced the expression of p-NF-*κ*B, and no significant difference between ODN/lip and mAb2G4/ODN/lip was observed (Figure 6(d)).

### 3.4. ODN/lip and mAb2G4/ODN/lip Elevated the Viability of H/R Cardiomyocytes In Vitro

MTT was applied to determine the viability of cardiomyocytes in each group after hypoxia/reoxygenation treatment. Results showed that hypoxia/reoxygenation treatment led to a decrease in cell viability (*P* < 0.0001). At the same time, ODN/lip could elevate the viability of H9c2 cardiomyocytes treated with hypoxia/reoxygenation. Experiments were conducted by adding a different dose of ODN/lip and mAb2G4/ODN/lip, and the results showed that the low amount of ODN/lip and mAb2G4/ODN/lip had a more significant effect (Figure 5(a)).

### 3.5. ODN/lip and mAb2G4/ODN/lip Inhibited the Activation of NF-*κ*B/NLRP3 Signaling Pathway in Hypoxia-Reoxygenation Cell Model

**Figure 7:**
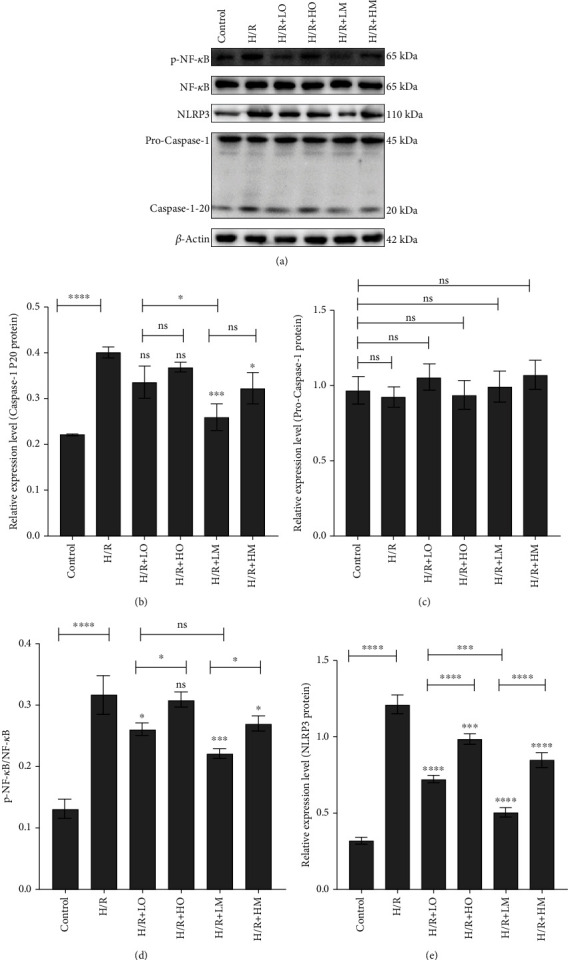
Analysis of the expression of inflammatory proteins by western blot in vitro. Results of western blot in the expression of caspase-1 P20 protein, procaspase-1 protein, p-NF-*κ*B, and NLRP3 protein in vitro (a). Effects of ODN/lip and mAb2G4/ODN/lip on the expression of caspase-1 P20 protein (b), procaspase-1 protein (c), p-NF-*κ*B (d), and NLRP3 protein (e) in H/R ventricular myocyte model. Control group (cells were cultured in the normal incubating condition), H/R group (cells were subjected to hypoxia for 2 hours and reoxygenation 1-hour treatment), LO group (treated with 2 *μ*g ODN/lip), HO group (treated with 4 *μ*g ODN/lip), LM group (treated with 2 *μ*g mAb2G4/ODN/lip), and HM group (treated with 4 *μ*g mAb2G4/ODN/lip). Data were expressed as mean ± S.D. ^ns^*P* > 0.05, ^∗^*P* < 0.05, ^∗∗^*P* < 0.01, ^∗∗∗^*P* < 0.005, and ^∗∗∗∗^*P* < 0.0001. H/R group vs. control group; LO group, HO group, LM group, and HM group vs. H/R group; LO group vs. HO group; LM group vs. HM group; LO group vs. LM group.

Western blot results showed that the expression of caspase-1 P20 protein, p-NF-*κ*B, and NLRP3 protein significantly increased in the H/R group compared with the control group (Figure 7(a)). But the difference in the expression of pro-caspase-1 protein between the H/R group and control group was not observed (Figure 7(c)), consistent with the results previously observed. No significant effect of ODN/lip on the expression of caspase-1-P20 protein was observed, while mAb2G4/ODN/lip could reduce the expression of caspase-1-P20 protein (Figure 7(b)). There was a significant increase in the expression of p-NF-*κ*B in the cell model of hypoxia/reoxygenation (*P* < 0.0001) (Figures 7(a) and 7(d)). Both ODN/lip and mAb2G4/ODN/lip showed inhibitory effects on NF-*κ*B expression, but the inhibitory effects between ODN/lip and mAb2G4/ODN/lip had no significant difference at the same dose (Figure 7(d)). In addition, the experimental results showed that low doses of ODN/lip and mAb2G4/ODN/lip had the most pronounced inhibitory effect on NF-*κ*B expression in hypoxic/reoxygenated cardiac myocytes (*P* < 0.05) (Figure 7(d)). The influence of ODN/lip and mAb2G4/ODN/lip on NLRP3 also fits well with the influence on NF-*κ*B expression (Figure 7(e)).

## 4. Discussions

The purpose of the present study is to investigate the effects of post-treatment of mAb2G4/ODN/lip in ameliorating myocardial ischemia-reperfusion injury and related mechanisms. In the present study, we evaluated the therapeutic effects of ODN-lip and mAb2G4/ODN/lip on the myocardial I/R injury model in vivo and the H/R injury model in vitro. Our findings revealed that ODN-lip and mAb2G4/ODN/lip exhibited protective effects against I/R injury of cardiomyocytes no matter in vivo or in vitro. In addition, mAb2G4/ODN/lip exerted more significant protective effects against I/R injury than ODN-lip in vivo experiments. In comparison, the increased beneficial effect of mAb2G4/ODN/lip in vitro experiments was not observed.

Neutrophils are heavily activated in the injured area and produce large amounts of oxygen radicals, which lead to myocardial plasma membrane disruption. And the damage rapidly extends to the entire cell causing structural disruption of myogenic fibers and mitochondrial damage [28, 29]. In line with these studies, the present results demonstrate that myocardial fibers appeared disrupted and discontinuous with deeper nuclear staining and irregular arrangement of myocardial cells accompanied by the infiltration of numerous neutrophils in the MI/RI group. The results of TTC staining proved our hypothesis, but the accuracy of staining results was easily influenced by some uncontrollable factors. For instance, the isolated myocardium cannot fully mimic the in vivo environment of rats, and Evans blue staining before TTC staining and sectioning after freezing could lead to partial inactivation of myocardial lactate dehydrogenase. In addition, staining by incubation at 37°C after freezing can lead to wrinkling of myocardial sections, which can affect the calculation of the myocardial infarct area. TNF-*α*, IL-6, IL-1*β*, LDH, and MDA increased in the MI/RI rats and were reduced by posttreatment of ODN-lip and mAb2G4/ODN/lip (Figures 3 and 5). Accumulated evidence shows that the expression of IL-1*β*, IL-6, and TNF-*α* increased a lot during inflammatory cardiac injury and was positively correlated with the degree of injury [30–32].

Accumulated studies suggested that mAb2G4 can bring liposomes to the surface of injured cells, thereby translocating ODN into the cytoplasm of damaged cardiomyocytes [21, 33]. Besides, the monoclonal anti-myosin antibody 2G4-attached cationic liposome without the NF-*κ*B decoy could exert protective effects on the injured cardiomyocytes. Related studies have shown that monoclonal antibodies and liposomes could combine to form a complex called immunoliposome. And the immunoliposome has been used to bind to the membrane of hypoxia-damaged cardiomyocytes to plug and seal the lesions in the blocked cell membrane, thereby reducing the ischemic and hypoxic damage of cardiac myocytes [34, 35]. We did not set the mAb2G4/liposome group in the present study because our results would not be influenced by the positive effect of mAb2G4/liposome on the injured cardiomyocytes. We constructed a hypoxia-reoxygenation model of mAb2G4/ODN/lip acting on H9C2 cardiomyocytes and explored the possible mechanism of hypoxia-reoxygenation protection produced by mAb2G4/ODN/lip treatment on cardiomyocytes, but whether the functions of the other organs would be influenced during the application of this complex requires deeper exploration. In the present study, we found that the mAb2G4/ODN/lip complex exerted more significant effects in protecting the hypoxic myocardium than ODN/lip in vivo experiment. However, the present results in the study were not sufficient to suggest that the mAb2G4/ODN/lip has more significant protective effects than ODN/lip in vitro. On the other hand, the results may suggest that the mAb2G4 does not change the pharmacological properties of ODN/lip. Given its character of safety and targetability, the mAb2G4 requires more extensive investigation to increase its application in clinical practice. Different doses of ODN were applied in the present study to determine whether the effect of ODN on NF-*κ*B is dose-related. Intriguingly, low doses of ODN-lip and mAb2G4/ODN/lip were significantly more effective than high doses of ODN-lip and mAb2G4/ODN/lip (Figures 5 and 7). It would be of interest to determine the specific mechanism causing that phenomenon. We speculate that the mechanism of that phenomenon may be related to the nonspecific reaction of excess cationic liposomes with cell membranes, causing cytosolic lysis and cell necrosis. The H9c2 cells undergoing hypoxia and reoxygenation were used to investigate the mechanism of action of mAb2G4/ODN/lip on the myocardium. However, it is conceivable that marked differences exist between myocardial cells in a working tissue in the presence of endothelial and blood cells which can participate in the inflammatory response and thus exacerbate the alterations following ischemia and reperfusion and resting isolated cells in culture. A more extensive study of the mechanism involved in the dose-related effects of mAb2G4/ODN/lip is necessary to fully solve this puzzle. The present results of this study suggest that we have much more to learn about the protective effects of the mAb2G4/ODN/lip on injured cardiomyocytes.

## 5. Conclusions

In conclusion, the results of our study provided sufficient evidence that mAb2G4/ODN/lip exerted cardioprotective effects on ischemic myocardium by improving cardiac cell viability, reducing the extent of cell damage, and depressing the NF-*κ*B-mediated inflammatory response. Thus, mAb2G4/ODN/lip may have potential therapeutic properties as a cardioprotective agent. And this research offers a novel strategy for patients with acute or unexpected ischemic heart diseases.

## Data Availability

The data that support the findings of this study are available from the corresponding authors upon reasonable request.

## Supplementary Materials

Supplementary Materials The supplementary file provides the full uncropped western blot gels of the proteins appeared in Figures 6(a) and 7(a).
