# Supplementary material for: Cardioprotection of mAb2G4/ODN/lip on Myocardial Ischemia-Reperfusion Injury via Inhibiting the NF-κB Signaling Pathway
Source: Cardiovasc Ther. 2023 Apr 27;2023:5034683. doi: 10.1155/2023/5034683 (PMC10159742; doi:10.1155/2023/5034683)

Supplementary data for FIGURE 5.

1. The full uncropped western blot gels of Actin.

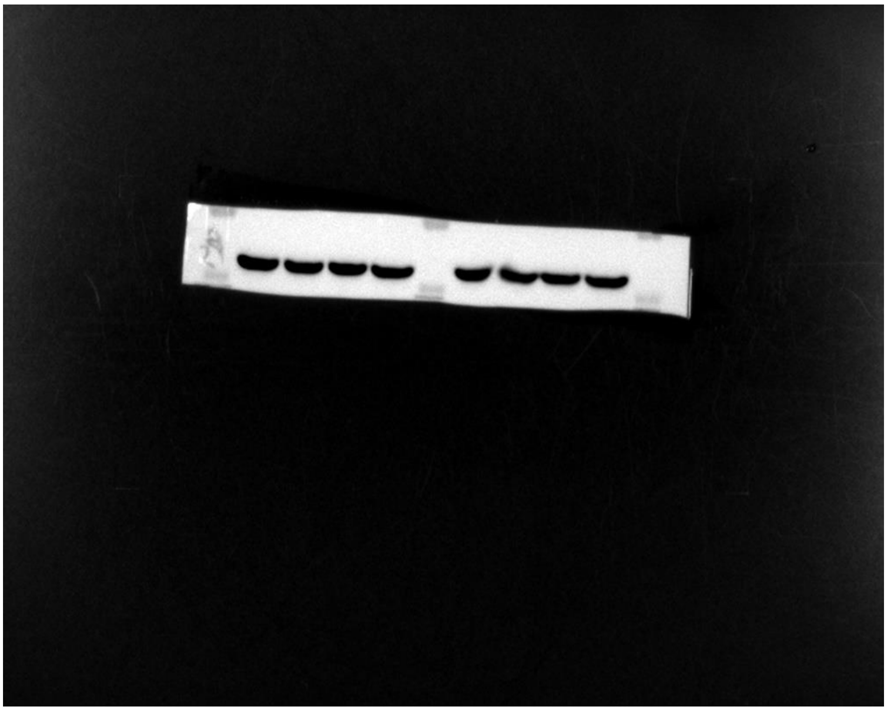

2. The full uncropped western blot gels of p-NF-κB

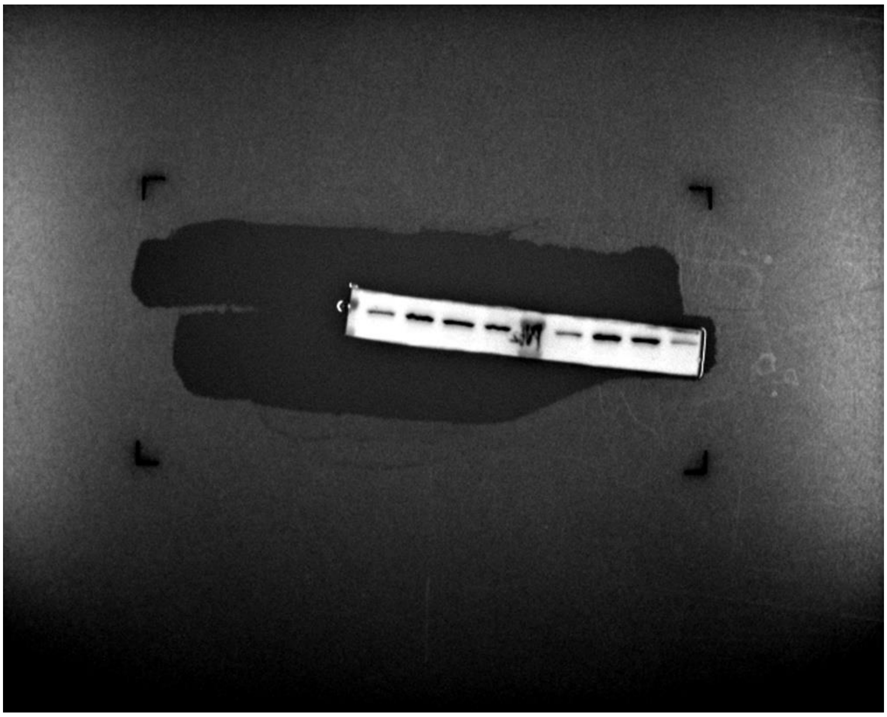

3. The full uncropped western blot gels of NLRP3

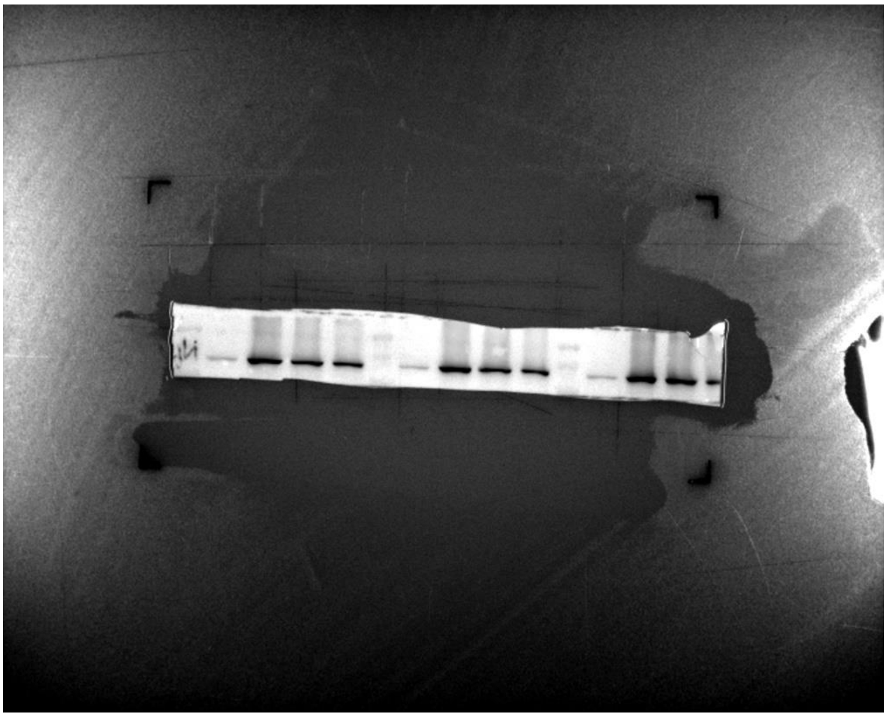

4. The full uncropped western blot gels of NF-κB

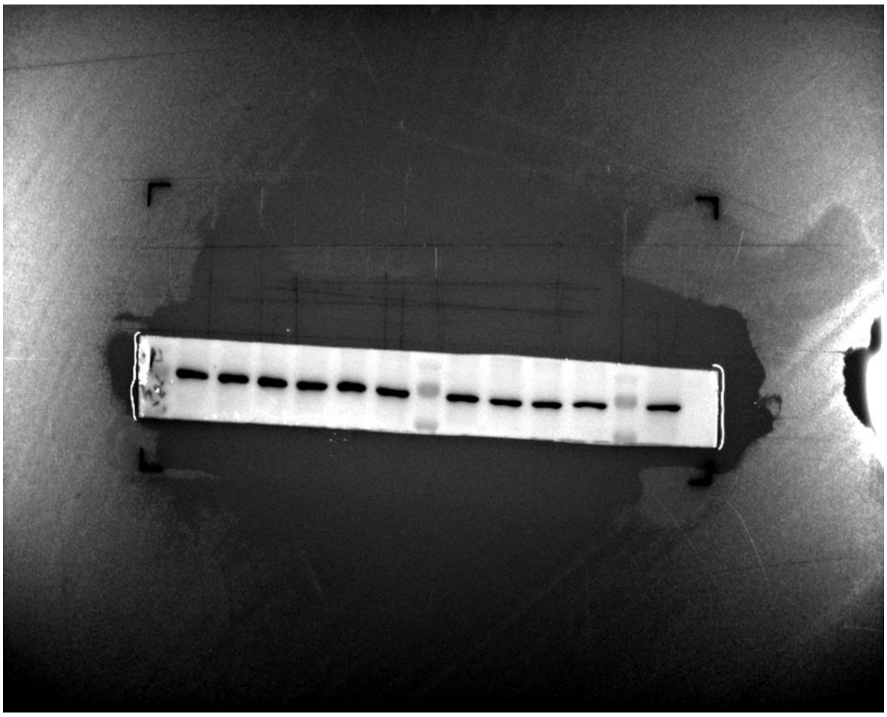

5. The full uncropped western blot gels of caspase

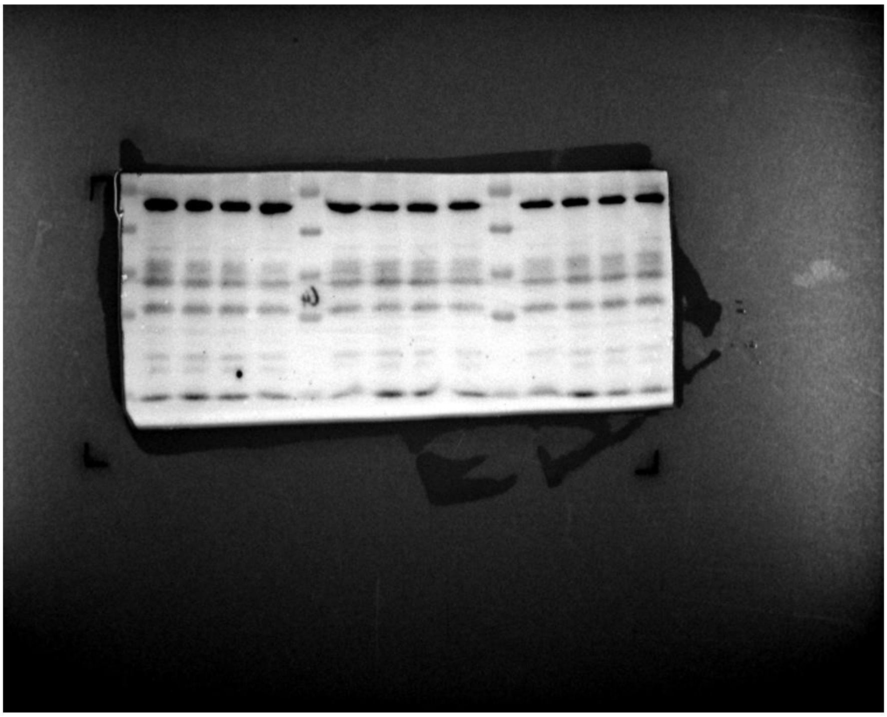

Supplementary data for FIGURE 7.

6. The full uncropped western blot gels of Actin.

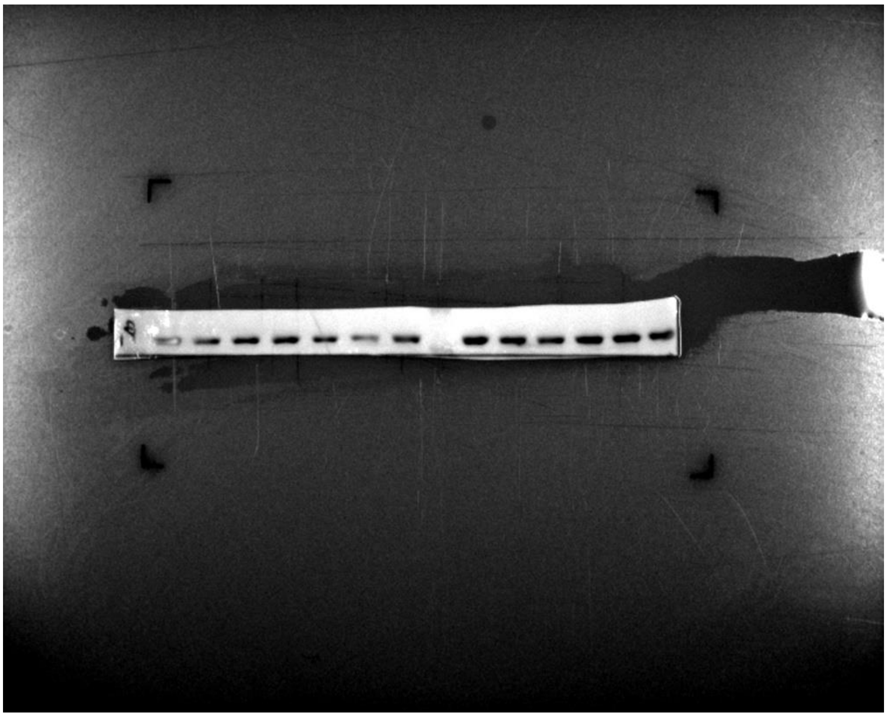

7. The full uncropped western blot gels of p-NF-κB

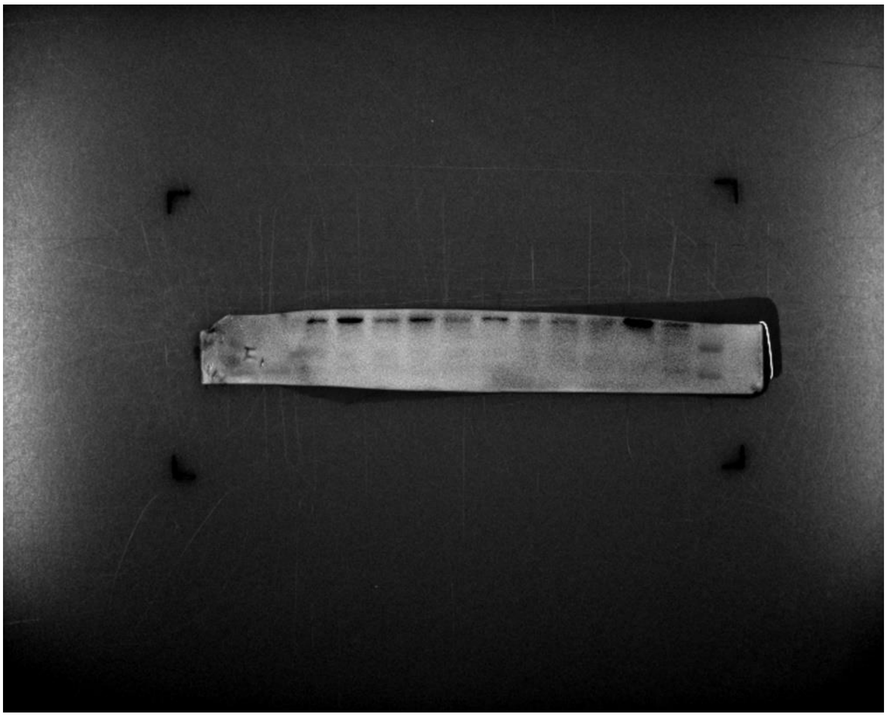

8. The full uncropped western blot gels of NLRP3

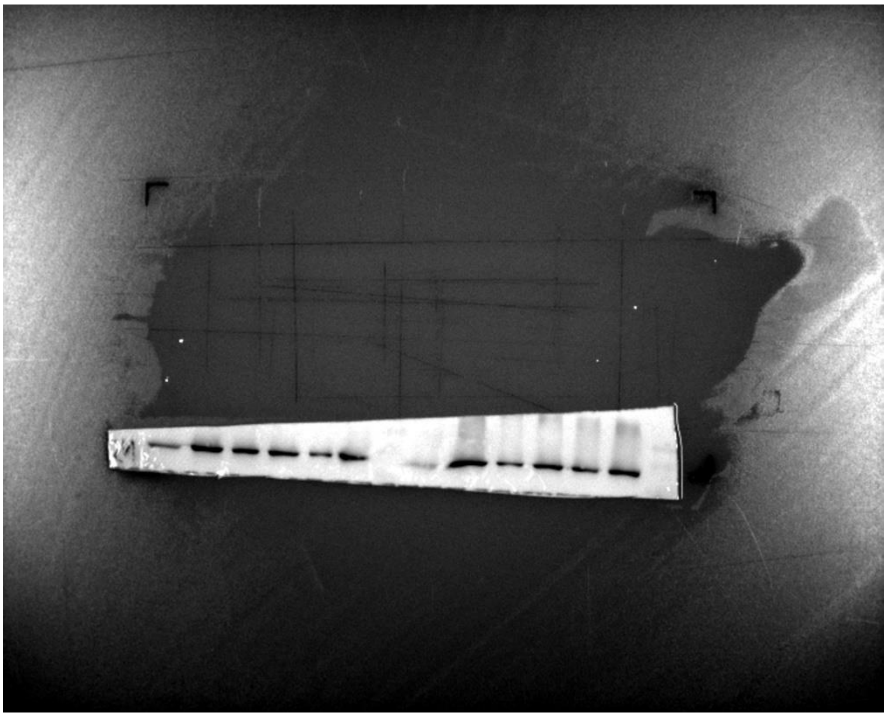

9. The full uncropped western blot gels of NF-κB

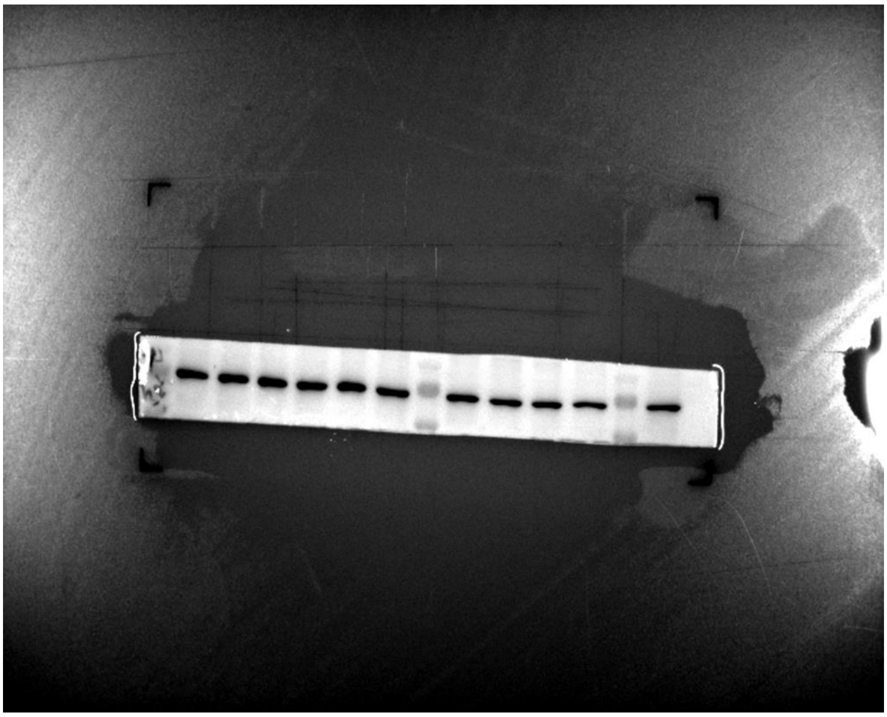

10. The full uncropped western blot gels of caspase

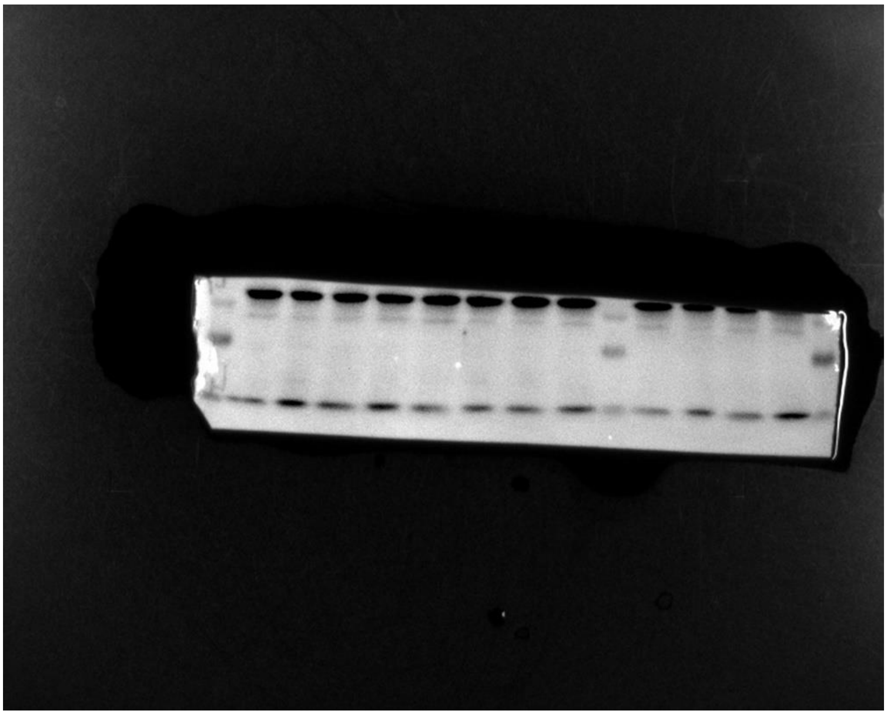

Supplement: Supplementary Materials — The supplementary file provides the full uncropped western blot gels of the proteins appeared in Figures 6(a) and 7(a). [file 5034683.f1.pdf]
